# Large Variation in Adherence to Diagnostic Guidelines in Hypertension Management in Swedish Primary Healthcare

**DOI:** 10.1111/jch.70079

**Published:** 2025-06-23

**Authors:** Mikko Hellgren, Kristina Bengtsson Boström, Katarina Hedin, Stefan Jansson, Staffan Nilsson, Gunnar Nilsson, Per Wändell, Patrik Wennberg

**Affiliations:** ^1^ University Health Care Research Centre, Örebro University Hospital Örebro Sweden; ^2^ School of Medical Sciences Örebro University Örebro Sweden; ^3^ School of Public Health and Community Medicine Institute of Medicine, Sahlgrenska Academy, University of Gothenburg Göteborg Sweden; ^4^ Futurum Region Jönköping County Jönköping Sweden; ^5^ Department of Health Medicine and Caring Sciences, Linköping University Linköping Sweden; ^6^ Department of Clinical Sciences in Malmö Family Medicine, Lund University Malmö Sweden; ^7^ Department of Public Health and Caring Sciences Uppsala University Uppsala Sweden; ^8^ Division of Family Medicine and Primary Care Department of Neurobiology Care Sciences and Society, Karolinska Institutet Huddinge Sweden; ^9^ Department of Public Health and Clinical Medicine Family Medicine, Umeå University Umeå Sweden

**Keywords:** blood pressure, guidelines, hypertension, primary care issues

## Abstract

High blood pressure (BP) is a frequent cause for visits to primary healthcare centers (PHCCs) in Sweden. Guidelines on methods for BP measurements for diagnosis of hypertension have recently been updated. We aimed to study adherence to diagnostic guidelines in hypertension management and evaluate whether adherence to guidelines was related to organizational or sociodemographic characteristics. Interviews with representatives from 76 randomly selected PHCCs from eight regions in Sweden were conducted. PHCCs’ use of 24‐h ambulatory BP monitoring (ABPM), home BP monitoring (HBPM) and BP measurements in both arms for the diagnosis of hypertension were chosen as proxy markers for adherence to diagnostic guidelines. An adherence index was created as a composite score of these diagnostic methods. The proportion of PHCCs stating they “often use” ABPM and HBPM were 13.7% and 16.0%, respectively, and 57.3% stated they performed BP measurements in both arms. Two PHCCs did not use ABPM, HBPM or BP measurements in both arms to diagnose hypertension. None of the organizational or sociodemographic characteristics (number of listed patients, Care Need Index (CNI), geographical location, ownership, investigation primarily led by doctor/nurse, dedicated team management, special training for hypertension and local routines) were associated with the adherence index. This study shows that adherence to diagnostic guidelines vary largely between PHCCs. No organizational characteristic, not even team‐based management, was associated with adherence to diagnostic guidelines. The variation raises questions about inequity healthcare and novel strategies that may be needed to improve PHCCs’ adherence to diagnostic guidelines in hypertension management. Trial Registration: ClinicalTrials.gov identifier: 263351 [www.researchweb.org]

**Abbreviations**
ABPM: 24‐h ambulatory blood pressure measurements
BP: blood pressure
GP: general practitioner
PHC: primary healthcare
PHCC: primary healthcare center

## 1 Introduction

Hypertension is a very common chronic disease with a prevalence that increases with age up to 50% [1, 2]. High blood pressure (BP) is also an important treatable risk factor for stroke, myocardial infarction, heart failure, and chronic kidney disease [3]. However, only about half of patients with hypertension achieve treatment goals [1]. In addition, there is a large proportion of undiagnosed patients [4, 5, 6, 7]. Despite this, there has been a decline in cardiovascular mortality for nearly four decades [8, 9]. Development in the field of hypertension has been dynamic in recent years, as shown by the changes in the European treatment guidelines (ESH/ESC 2018 and 2023) [10, 11]. The ESH guidelines recommend that the diagnosis of hypertension be based on office BP measurements. Access to 24‐h ambulatory BP measurement (ABPM) and home BP measurements (HBPM) can be useful and is also recommended in various situations [12, 13]. The new guidelines present new approaches to diagnostics, with choices of measuring equipment, risk assessment, and hypertension treatment.

Healthcare in Sweden is managed by 21 politically highly autonomous regions, and healthcare is financed primarily through taxes with a minor contribution from patient fees. Primary healthcare centers (PHCCs) can be both private and public driven, although both are financed by taxes within each region. In general, primary healthcare (PHC) in Sweden is challenged by a low continuity of care and decreased public trust [14]. The current demographic trend, with an increasing number of elderly individuals, will increase the workload in PHCCs and necessitate organizational changes [15, 16]. Since hypertension is both very common and important for both primary and secondary prevention, efficient, high‐quality care for this patient group is crucial. One example of organizational changes in PHCCs is task shifting. In Sweden there have been successful examples, such as nurse‐led management utilizing algorithms to escalate treatment and systematic follow‐up of the BP‐lowering impact and management of side effects, which showed postponement of stroke at follow‐up [17]. In addition, increasing treatment responsibility has been transferred to the patient, for example, in the form of HBPM or self‐measurement in a separate room at the PHCCs [18]. In evaluations, HBPM has been shown to be superior to BP taken in the office and as good as ABPM for use in diagnosis and follow‐up [19, 20]. Furthermore, guidelines emphasize the importance of checking BP in both arms at diagnosis, as differences in BP between arms could be related to atherosclerosis and increased risk for stroke [10, 11, 21, 22].

The aim of this study was to study adherence to diagnostic guidelines in hypertension management in PHC, and as a secondary aim, we sought to evaluate whether team‐based management or other organizational or sociodemographic characteristics were associated with adherence to diagnostic guidelines regarding hypertension.

## 2 Materials and Methods

### 2.1 Study Population and Data Collection

In this study, data were collected from eight participating regions (Jämtland‐Härjedalen, Jönköping, Stockholm, Värmland, Västerbotten, Västra Götaland, Örebro, and Östergötland). PHCCs from these regions were selected using a random number generator. In total, 76 interviews were conducted with one employee from each PHCCs using a structured questionnaire between December 2019 and January 2021. The manager of the PHCC selected a coworker with the appropriate knowledge of the management of care of hypertension in the PHCC. Of the 76 interviews, there were 35 interviews with a physician and 39 interviews with a nurse, one interview with a care administrator in a manager position, and one interview with an assistant nurse in a manager position. One PHCC had missing answers regarding the adherence index and was not included in the statistical analysis. The answers were typed by the authors into Smart‐Trials (SMART‐TRIAL 2019 version 2.0 ApS), a data repository managed by the University of Örebro. More details about the participants and data collection in this study can be found in a previously published paper [23].

### 2.2 BP Measurements

The use of ABPM and HBPM was reported as “never,” “sometimes,” and “often,” and the use of BP measurement in both arms was reported as “yes” or “no.”

### 2.3 Organizational and Sociodemographic Characteristics

PHCCs were categorized geographically based on their regional location. Furthermore, PHCC locations were defined as rural according to a study‐specific definition of having less than 10,000 inhabitants and being more than 20 km from an emergency department. CNI (Care Need Index) is a region‐adjusted index measuring health status in the population and was used as a sociodemographic variable. For each PHCC, the number of listed patients and organizational form (public or private/other forms) were assessed. To evaluate staff workload and proficiency, patients per tenured general practitioner (GP), the tenured GP ratio (number of tenured GPs divided by all medical doctors), and the number of patients per nurse were included in the analysis. The routine and management of hypertension in each PHCC were evaluated based on whether they had a dedicated team to manage hypertension, staff with special training for hypertension management, followed specific routines for hypertension management, and which staff members primarily investigated patients with hypertension.

### 2.4 Proxy Markers for Adherence and Adherence Index

To analyze the association between organizational and sociodemographic characteristics and adherence, an index for adherence to diagnostic guidelines was constructed based on three proxy markers for adherence to the use of recommended approaches for diagnostic BP measurement in ESC/ESH Guidelines: ABPM, HBPM, and measurement in both arms. A high adherence index score indicates a higher adherence to diagnostic guidelines (Table 1). As there were few units with the lowest and highest scores on the adherence index, the PHCCs were grouped into three categories (0–2 points, 3 points and 4–5 points) and in two categories (0–2 points and 3–5 points) for a complementary check regarding results (Table 1, Table S1).

**TABLE 1:** Distribution of ambulatory blood pressure, home blood pressure measurements, and measurements in both arms at diagnosis and adherence index among 76 primary healthcare centers in eight regions in Sweden.

	*n* (%)
Ambulatory BP	
Never (0 points)	2 (2.7)
Sometimes (1 point)	50 (66.7)
Often (2 points)	23 (30.7)
Home blood pressure	
Never (0 points)	25 (33.3)
Sometimes (1 point)	38 (50.7)
Often (2 points)	12 (16.0)
Both arms	
No (0 point)	32 (42.7)
Yes (1 point)	43 (57.3)
PHCCs	

Total (*n*)	Adherence index Average score	SD	95% CI
75	2.68	1.08	2.44–2.92
Adherence index merged into three groups			
0–2	29 (38.7)		
3	30 (40.0)		
4–5	16 (21.4)		
Adherence index merged into two groups			
0–2	29 (38.7)		
3–5	46 (61.4)		

*Note*: One primary healthcare center was excluded from the analysis due to missing answers.

### 2.5 Statistical Analyses

For comparison between groups, the Mantel–Haenszel Chi‐square test was used for ordered categorical variables, and the Chi‐square test was used for non‐ordered categorical variables. The Jonckheere–Terpstra test was used for continuous variables. The statistical analysis was performed in SAS 9.4 (SAS Institute, Cary, NC, USA).

## 3 Results

**FIGURE 1:**
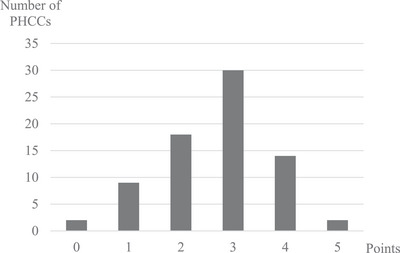
Adherence index scores (from zero to five) for 76 primary healthcare centers in eight regions in Sweden. Adherence index is based on the frequency of ambulatory blood pressure measurements (never, sometimes, often), home blood pressure measurements (never, sometimes, often), and measurements in both arms (no, yes). Data on diagnostic blood pressure measurements were missing from one PHCC. PHCCs, primary healthcare centers.

Mean listed patients and mean number of patients in relation to staff in our study were: 9199 listed patients, 3199 patients per tenured GP, and 1234 listed patients per nurse (Table 2). The majority, 77.3% (*n* = 58) of PHCCs were public‐driven (Table 2). A total of 8% (*n* = 6) PHCCs had no tenured GPs, and 16% (*n* = 12) had a single tenured GP, and most of these PHCCs had few listed patients and were in rural areas of Sweden. Half of the interviewed PHCCs (*n* = 38, 50%) stated that they had a team of professionals dedicated to the treatment of hypertension (defined as “dedicated team”); these teams were predominantly managed by nurses (*n* = 31 of 38, 82%). The proportion of PHCCs staff who stated that their PHCC often used ABPM and HBPM was 13.7% and 16.0%, respectively, and 57.3% stated they performed BP measurements in both arms (Table 1). All but two stated they used ABPM sometimes or often at diagnosis. Two stated they did not use ABPM, HBPM, or BP measurements in both arms to diagnose hypertension. The adherence index score was normally distributed, and the average was 2.68 (95% CI: 2.44–2.92) (Figure 1). Only two PHCCs achieved the highest score on the adherence index. None of the organizational or sociodemographic characteristics were associated with the adherence index (Table 2). To increase the robustness of the analysis, the adherence index was tested with two groups (Table S1), and the *p* values only changed slightly.

**TABLE 2:** Characteristics of primary healthcare centers and adherence index scores divided into three groups (0–2, 3, and 4–5) for 76 primary healthcare centers in eight regions in Sweden.

	Total (*n* = 75)	0–2 (*n* = 29)	3 (*n* = 30)	4–5 (*n* = 16)	*p* value
**Listed patients**					
Mean (SD)	9199 (4934)	8219 (3839)	9848 (4841)	9759 (6653)	0.63
Median (Min; Max)	9000 (763; 31 350)	9099 (763; 16 000)	9518 (2656; 24 000)	7998 (2206; 31 350)	
Number *n*	*n* = 75	*n* = 29	*n* = 30	*n* = 16	
**Patients per tenured GP**					
Mean (SD)	3199 (1955)	3433 (2269)	3245 (1966)	2719 (1291)	0.47
Median (Min; Max)	2470 (1300; 11 300)	2553 (1300; 10 770)	2567 (1625; 11 300)	2206 (1599; 6693)	
Number *n* ^a^	*n* = 69	*n* = 25	*n* = 29	*n* = 15	
**Patients per nurse**					
Mean (SD)	1234 (558)	1134 (418)	1249 (506)	1386 (817)	0.38
Median (Min; Max)	1100 (429; 3675)	1077 (533; 2058)	1107 (429; 2400)	1179 (483; 3675)	
Number *n*	*n* = 75	*n* = 29	*n* = 30	*n* = 16	
**Tenured GP ratio**					0.69
Mean (SD)	0.697 (0.320)	0.645 (0.357)	0.750 (0.294)	0.693 (0.301)	
Median (Min; Max)	0.8 (0; 1)	0.75 (0; 1)	0.854 (0; 1)	0.75 (0; 1)	
Number *n*	*n* = 75	*n* = 29	*n* = 30	*n* = 16	
**CNI**					
Mean (SD)	1.55 (0.74)	1.63 (0.76)	1.49 (0.58)	1.50 (0.99)	0.27
Median (Min; Max)	1.25 (0.66; 4.55)	1.26 (0.78; 3.54)	1.36 (0.66; 2.48)	1.1 (0.67; 4.55)	
Number *n*	*n* = 75	*n* = 29	*n* = 30	*n* = 16	
**Location in a rural area**					
No	51 (68.0%)	21 (72.4%)	20 (66.7%)	10 (62.5%)	
Yes	24 (32.0%)	8 (27.6%)	10 (33.3%)	6 (37.5%)	0.48
**Ownership**					
Public	58 (77.3%)	23 (79.3%)	23 (76.7%)	12 (75.0%)	
Private/Other forms	17 (22.7%)	6 (20.7%)	7 (23.3%)	4 (25.0%)	0.73
Örebro	10 (13.3%)	5 (17.2%)	4 (13.3%)	1 (6.3%)	
Västerbotten	7 (9.3%)	3 (10.3%)	3 (10.0%)	1 (6.3%)	
Stockholm	10 (13.3%)	1 (3.4%)	6 (20.0%)	3 (18.8%)	
Västra Götaland	10 (13.3%)	4 (13.8%)	2 (6.7%)	4 (25.0%)	
Östergötland	9 (12.0%)	3 (10.3%)	4 (13.3%)	2 (12.5%)	
Jämtland/Härjedalen	9 (12.0%)	5 (17.2%)	1 (3.3%)	3 (18.8%)	
Värmland	10 (13.3%)	6 (20.7%)	4 (13.3%)	0 (0.0%)	
Jönköping	10 (13.3%)	2 (6.9%)	6 (20.0%)	2 (12.5%)	0.33
**Dedicated team management HT**					
No	37 (49.3%)	15 (51.7%)	16 (53.3%)	6 (37.5%)	
Yes	38 (50.7%)	14 (48.3%)	14 (46.7%)	10 (62.5%)	0.43
**Who investigates HT**					
Primarily GPs	48 (64.0%)	18 (62.1%)	21 (70.0%)	9 (56.3%)	
Primarily Nurses	19 (25.3%)	9 (31.0%)	6 (20.0%)	4 (25.0%)	
Dedicated staff for HT/Cardiac diseases	8 (10.7%)	2 (6.9%)	3 (10.0%)	3 (18.8%)	0.66
**Special training for HT management**					
No	40 (53.3%)	18 (62.1%)	16 (53.3%)	6 (37.5%)	
Yes	35 (46.7%)	11 (37.9%)	14 (46.7%)	10 (62.5%)	0.12
**Local/Regional routines for management of HT**					
No	63 (84.0%)	23 (79.3%)	26 (86.7%)	14 (87.5%)	
Yes	12 (16.0%)	6 (20.7%)	4 (13.3%)	2 (12.5%)	0.43

*Note*: Data are presented in absolute numbers (*n*) and percentages (%) for each answer. The adherence index scores are divided into categories: 0–2, 3, and 4–5 points. For categorical variables, *n* (%) is presented, while for continuous variables, means (SD) and medians (min; max) are presented.

^a^Six PHCCs lacked any tenured GPs and were therefore omitted from the analysis. One PHCC was excluded from the analysis due to missing answers to the questions about ambulatory blood pressure, home blood pressure, or measurement in both arms.

## 4 Discussion

This study showed that approaches for diagnostic BP measurement and adherence to diagnostic guidelines vary greatly among PHCCs in Sweden. However, few PHCCs received the lowest or highest scores on the adherence index. The variance between PHCCs could not be explained by any of the studied organizational or sociodemographic variables.

Half of the PHCCs stated that they measure BP in both arms, about two‐thirds stated that they sometimes or often collect HBPM, and all but two stated that they sometimes or often measure ABPM prior to diagnosing hypertension. Only two PHCCs achieved the highest score on the adherence index, but it could be argued that both HBPM and ABPM are not essential for diagnosis but rather complementary to rule out white‐coat HT or find masked HT. However, from a patient perspective, concerning engagement and knowledge, both HBPM and ABPM could be beneficial.

PHC in Sweden is characterized by a low continuity of care, and many PHCCs lack tenured GPs, a factor that is known to affect the quality of care in PHCs [24, 25]. It has long been known that continuity of care is appreciated by patients [26, 27]. However, in our study, we could not observe any significant differences in adherence to guidelines based on listed patients, tenured GP ratio, patients per tenured GP, or patients per nurse.

Overall, in Swedish PHC, the management of chronic conditions is trending toward task shifting from physicians to other professions and team‐based approaches. For example, healthcare teams have existed for a long time for children and patients with diabetes and more recently for the elderly, patients with hypertension, cardiac failure, chronic pain, and psychiatric disorders. Nurses and assistant nurses often play an important role in these teams and can increase the effectiveness of care [28]. In this study, the association between a large set of background factors and an adherence index was analyzed. Surprisingly, not even the existence of a specialized team for hypertension management at the PHC was associated with adherence to diagnostic guidelines. It has been shown that the management of hypertension differs between PHCCs [23], along with the attainment of BP targets (from less than 40% to more than 85%, average 60% [29]. The lack of significant findings regarding explanatory factors in the current study and the variation in the achievement of BP goals in previous research leads to the conclusion that adherence to guidelines in hypertension management is a multifaceted and complex issue. This finding is in line with conclusions drawn from a recent qualitative study that also highlighted the complicated nature of factors behind a suboptimal adherence to guidelines [30].

### 4.1 Strengths and Limitations

The strengths of the study include that it covered a large area of Sweden and that the PHCCs were randomly selected. Moreover, a comprehensive set of organizational and demographic background variables was collected, and a sensitivity analysis was conducted. An important limitation is that data in the study were self‐reported, which could lead to items being both over‐ and underestimated. From each PHCCs, one selected co‐worker was interviewed, and the outcome of this survey might have been different if several professionals at each PHCCs were interviewed. Participation in the study could also be biased toward the PHCCs more interested in hypertension care than the average PHCC. Since patient data like BP levels and efforts to increase adherence to lifestyle recommendations were not part of this study, no general conclusions on patient outcomes can be drawn based on these interviews.

## 5 Conclusions

The main findings in this study were that it showed a large variation in adherence to diagnostic guidelines for hypertension. Since none of the evaluated organizational or sociodemographic characteristics was associated with the adherence index, including the existence of a specialized team for hypertension management at the PHCC, novel strategies such as continuity in care or continuous evaluation of treatment goals comparing PHCCs may need to be developed to improve adherence to diagnostic guidelines in hypertension management.

## Ethics Statement

Ethical approval was applied for and granted by the Swedish Ethical Review Authority (registration number 2019‐0169), with an amendment for the addition of regions (2020‐00478).

## Consent

No patients were included in the study. Written consent was obtained from all the interviewed professionals in the study.

## Conflicts of Interest

The authors declare no conflicts of interest.

## Supporting information




**Supplementary Table S1**: Characteristics of primary health care centres and adherence index scores divided into two groups (0‐2, 3‐5) for 76 primary health care centres in eight regions in Sweden.

## Data Availability

The data that support the findings of this study are available upon reasonable request to the corresponding author. This study is registered in Research web (ww.researchweb.org) project number 263351, created 2019‐02‐20.
