# Supplementary material for: Large Variation in Adherence to Diagnostic Guidelines in Hypertension Management in Swedish Primary Healthcare
Source: J Clin Hypertens (Greenwich). 2025 Jun 23;27(6):e70079. doi: 10.1111/jch.70079 (PMC12185900; doi:10.1111/jch.70079)
Supplement: Supplementary file 1 — Supplementary Table S1: Characteristics of primary health care centres and adherence index scores divided into two groups (0‐2, 3‐5) for 76 primary health care centres in eight regions in Sweden. [file JCH-27-e70079-s001.docx]

Supplementary Table 1. Characteristics of primary health care centres and adherence index scores divided into two groups (0–2, 3–5) for 76 primary health care centres in eight regions in Sweden.

|  | **Total (n=75)** | **0-2 (n=29)** | **3-5 (n=46)** | **p-value** |
| --- | --- | --- | --- | --- |
| **Listed patients**  Mean (SD)  Median (Min;Max)  Number n | 9199 (4934) 9000 (763; 31350) n=75 | 8219 (3839) 9099 (763; 16000) n=29 | 9848 (4841) 9518 (2656; 24000) n=30 | 0.45 |
| **Patients per tenured GP**  Mean (SD)  Median (Min;Max)  Number n^a^ | 3199 (1955)  2470 (1300; 11300)  n=69 | 3433 (2269)  2553 (1300; 10770)  n=25 | 3065 (1768)  2430 (1599; 11300)  n=44 | 0.89 |
| **Patients per nurse**  Mean (SD)  Median (Min;Max)  Number n | 1234 (558) 1100 (429; 3675) n=75 | 1134 (418) 1077 (533; 2058) n=29 | 1297 (626)  1125 (429; 3675)  n=46 | 0.40 |
| **Tenured GP ratio**  Mean (SD)  Median (Min;Max)  Number n | 0.697 (0.320)  0.8 (0; 1)  n=75 | 0.645 (0.357)  0.75 (0; 1)  n=29 | 0.730 (0.294)  0.8 (0; 1)  n=46 | 0.40 |
| **CNI**  Mean (SD)  Median (Min;Max)  Number n | 1.55 (0.74) 1.25 (0.66; 4.55) n=75 | 1.63 (0.76) 1.26 (0.78; 3.54) n=29 | 1.50 (0.74)  1.23 (0.66; 4.55)  n=46 | 0.39 |
| **Location in rural area** |  |  |  |  |
| No | 51 (68.0%) | 21 (72.4%) | 30 (65.2%) |  |
| Yes | 24 (32.0%) | 8 (27.6%) | 16 (34.8%) | 0.52 |
| **Ownership** |  |  |  |  |
| Public | 58 (77.3%) | 23 (79.3%) | 35 (76.1%) |  |
| Private/other forms | 17 (22.7%) | 6 (20.7%) | 11 (23.9%) | 0.75 |
| **Included regions** |  |  |  |  |
| Örebro | 10 (13.3%) | 5 (17.2%) | 5 (10.9%) |  |
| Västerbotten | 7 (9.3%) | 3 (10.3%) | 4 (8.7%) |  |
| Stockholm | 10 (13.3%) | 1 (3.4%) | 9 (19.6%) |  |
| Västra Götaland | 10 (13.3%) | 4 (13.8%) | 6 (13.0%) |  |
| Östergötland | 9 (12.0%) | 3 (10.3%) | 6 (13.0%) |  |
| Jämtland/Härjedalen | 9 (12.0%) | 5 (17.2%) | 4 (8.7%) |  |
| Värmland | 10 (13.3%) | 6 (20.7%) | 4 (8.7%) |  |
| Jönköping | 10 (13.3%) | 2 (6.9%) | 8 (17.4%) | 0.28 |
| **Dedicated team management HT** |  |  |  |  |
| No | 37 (49.3%) | 15 (51.7%) | 22 (47.8%) |  |
| Yes | 38 (50.7%) | 14 (48.3%) | 24 (52.2%) | 0.74 |
| **Who investigates HT** |  |  |  |  |
| Primarily GPs | 48 (64.0%) | 18 (62.1%) | 30 (65.2%) |  |
| Primarily Nurses | 19 (25.3%) | 9 (31.0%) | 10 (21.7%) |  |
| Dedicated staff for  HT/cardiac diseases | 8 (10.7%) | 2 (6.9%) | 6 (13.0%) | 0.53 |
| **Special training HT management** |  |  |  |  |
| No | 40 (53.3%) | 18 (62.1%) | 22 (47.8%) |  |
| Yes | 35 (46.7%) | 11 (37.9%) | 24 (52.2%) | 0.23 |
| **Local/regional routines for management of HT** |  |  |  |  |
| No | 63 (84.0%) | 23 (79.3%) | 40 (87.0%) |  |
| Yes | 12 (16.0%) | 6 (20.7%) | 6 (13.0%) | 0.38 |

Data are presented in absolute numbers (n) and (%) for each answer. Adherence index scores were divided into categories: 0–2 and 3–5 points. For categorical variables, n (%) is presented. For continuous variables, means (SD) and medians (min; max) are presented. ^a^ Six PHCCs lacked any tenured GPs and were therefore omitted from the analysis. One PHCC was excluded from analysis due to missing answers to the questions about ambulatory BP, home blood pressure or measurement in both arms.
